# Correction: Trends and determinants of antenatal care use and quality in Bangladesh: Insights from demographic and health survey data

**DOI:** 10.1371/journal.pone.0347391

**Published:** 2026-04-15

**Authors:** The PLOS One Staff

## Notice of Republication

This article was republished on April 2, 2026, to correct an error in the author list. Md. Abdul Quayyum, Md. Goffar Hossain, and Md. Akhtarul Islam were erroneously displayed as Abdul Quayyum, Goffar Hossain, and Akhtarul Islam, respectively. The correct citation is: Barna SD, Quayyum MA, Hossain MG, Islam MA, Rahman F (2025) Trends and determinants of antenatal care use and quality in Bangladesh: Insights from demographic and health survey data. PLoS One 20(11): e0337449. https://doi.org/10.1371/journal.pone.0337449. The publisher apologizes for the error. Please download this article again to view the correct version. The originally published, uncorrected article and the republished, corrected articles are provided here for reference.

## Supporting information

S1 File Originally published, uncorrected article. (PDF)

S2 File Republished, corrected article. (PDF)
