# Supplementary material for: Correction: Trends and determinants of antenatal care use and quality in Bangladesh: Insights from demographic and health survey data
Source: PLoS One. 2026 Apr 15;21(4):e0347391. doi: 10.1371/journal.pone.0347391 (PMC13082617; doi:10.1371/journal.pone.0347391)
Supplement: S2 File — (PDF) [file pone.0347391.s002.pdf]

PLOS One

RESEARCH ARTICLE

Trends and determinants of antenatal care use and quality in Bangladesh: Insights from demographic and health survey data

Sutapa Dey Barna*, Md. Abdul Quayyum, Md. Goffar Hossain, Md. Akhtarul Islam, Fuad Rahman

Statistics Discipline, Science Engineering and Technology School, Khulna University, Khulna, Bangladesh

* sutapa@stat.ku.ac.bd

Check for updates

OPEN ACCESS

Citation: Barna SD, Quayyum MA, Hossain MG, Islam MA, Rahman F (2025) Trends and determinants of antenatal care use and quality in Bangladesh: Insights from demographic and health survey data. PLoS One 20(11): e0337449. https://doi.org/10.1371/journal.pone.0337449

Editor: Muhammad Haroon Stanikzai, Kandahar University, Faculty of Medicine, AFGHANISTAN

Received: March 23, 2025

Accepted: November 6, 2025

Published: November 24, 2025

Copyright: © 2025 Barna et al. This is an open access article distributed under the terms of the Creative Commons Attribution License, which permits unrestricted use, distribution, and reproduction in any medium, provided the original author and source are credited.

Data availability statement: "Third party data was obtained for this study from The DHS Program (https://dhsprogram.com/). Data may be requested from The DHS Program after creating an account and submitting a concept note. More access information can be found on

Abstract

Background

High-quality antenatal care (ANC) reduces maternal and infant mortality and improves health outcomes, particularly in low-income countries. To assess the quality of ANC, three criteria are used: the number of visits, the timing of care initiation, and the inclusion of all recommended care components. The goal of this study was to identify and compare the factors associated with attending 4+ and 8+ANC visits as well as obtaining high-quality ANC.

Methods

Data from the Bangladesh Demographic and Health Survey (BDHS) 2017--2018 and 2022 were used to evaluate the prevalence of ANC, and a binary logistic regression model was used. High quality ANC included the following components: blood pressure measurement, urine tests for detecting bacteriuria and proteinuria, blood tests for syphilis and anemia, iron supplementation, vitamin A administration, a child's health check before discharge, and health education.

Results

In 2017--2018, 48% of women attended four or more visits, which decreased to 41% in 2022. ANC attendance for eight or more visits declined from 12% to 5%, and approximately 8% of women received no ANC in both periods. Higher levels of education among mothers and their spouses, urban residency and higher household wealth were positively correlated with ANC attendance and quality. Notably, essential components such as blood pressure monitoring and iron supplementation were more common among women with higher socioeconomic status, but their prevalence declined between 2017–2018 and 2022.

The DHS Program website (https://dhsprogram.com/data/Access-Instructions.cfm). The data set is openly available upon permission from the MEASURE DHS website (https://www.dhsprogram.com/data/available-datasets.cfm). The authors confirm that interested researchers would be able to access these data in the same manner as the authors. The authors also confirm that they had no special access privileges that others would not have".

Funding: The author(s) received no specific funding for this work.

Competing interests: The authors have declared that no competing interests exist.

Conclusions

Continuous efforts must be made in Bangladesh to improve access to higher-quality ANC. It is vital to target women with lower levels of education who come from low-income families. More focus must be placed on enhancing women's education for long-term improvement.

Introduction

Since pregnancy is a crucial time in a woman's life, the provision of high-quality ANC is essential to ensure maternal and fetal health while also providing an opportunity to identify high-risk women for screening, prevention, and treatment of cardiovascular disease, the leading cause of morbidity and mortality [1]. In 2021, global maternal deaths reached 267,000, a 40% decline in MMR from 328 per 100,000 live births in 2000–197, yet progress stalled post-2020 due to pandemic disruptions and inequities. Disproportionately affecting low- and lower-middle-income countries (94% of cases), where one woman dies every two minutes from preventable causes, this underscores the urgent need for accelerated investments in equitable maternal health services to achieve SDG targets by 2030 [2]. It has been hard to lower the number of mothers who die. For instance, the number of maternal fatalities around the world only went down by 2.3% each year from 1990 to 2015. This shows that development was consistent but not enough to fulfill global goals [3]. Bangladesh, as a developing country, faces significant challenges in maternal healthcare. The maternal mortality ratio in Bangladesh remains high, contributing to the country's overall health burden. In 2017, the maternal mortality rate in Bangladesh was 173, a decrease of 6.99% from 2016 [4]. In the examined sub-Saharan African countries, the prevalence of at least eight ANC visits ranged from a high of 98.7% in Zambia to a low of 73.4% in Libya [5].

The World Health Organization (WHO) recommends initiating ANC within the first trimester and attending at least eight visits during an uncomplicated pregnancy to optimize maternal and neonatal outcomes [5,6]. In addition to the frequency of visits, the quality of ANC is determined by the timing of the first visit and the inclusion of essential components such as blood pressure measurement, urine and blood tests, iron-folic acid supplementation, tetanus toxoid immunization, and health education [7–9]. Despite these guidelines, access to comprehensive ANC remains limited in Bangladesh, particularly among women from rural and low-income backgrounds [10]. IFA supplementation and TT immunization during pregnancy decreased the risk of infant mortality in Bangladesh. Baseline proteinuria was substantially associated with increased rates of preeclampsia, preterm birth, and growth restriction in expectant women with treated chronic hypertension, even at proteinuria values previously considered to be within the normal range (less than 300 mg/d) [11]. The effect of the WHO's recommended number of prenatal care visits for developing countries on birth outcomes is highly robust to changes in birth outcome measures but is significant only in urban areas [12]. This may be due to the inferior quality of prenatal care

services received in rural areas. A lack of access to health providers and facilities has contributed to nearly three-quarters (73%) of mothers in Bangladesh not receiving four or more ANC visits from trained health professionals, let alone the eight visits that the WHO recently recommended [13]. In comparison, only 49% of women living in rural areas receive ANC from a skilled practitioner, whereas 74% of women living in urban areas do [14]. Joshi et al. (2014) reported that only 50% of Nepalese women attended the recommended minimum of four prenatal appointments during their previous pregnancy, indicating possible gaps in ANC usage [15].

Persistent pro-rich inequities and a pronounced rural-urban divide continue to hinder equitable access to high-quality ANC services in Bangladesh [16]. Although national surveys such as the BDHS report gradual improvements in ANC coverage, significant gaps remain in both utilization and service quality, particularly among women from rural and disadvantaged communities. Understanding the determinants of ANC utilization and quality is essential for designing targeted policy interventions aimed at achieving Sustainable Development Goal (SDG) 3, which aims to reduce the global maternal mortality ratio to less than 70 per 100,000 live births by 2030 [17].

The primary objective of this population-based study, therefore, is to investigate the factors associated with the use and quality of ANC in Bangladesh and analyze high-risk factors and complications linked to maternal mortality. This study, which utilizes data from BDHS 2018 and 2022, explores the factors associated with ANC usage and quality in Bangladesh, with a focus on identifying barriers to reaching the WHO-recommended eight ANC visits. The findings of this trend analysis will be useful in health policy to respond to deficiencies in ANC service delivery, especially in rural unidentified and less served mother and newborn communities. The purpose of this research is to provide some recommendations for understanding the trend of ANC usage and potential predictors of ANC usage and service quality in Bangladesh.

Method

Data sources

This study used survey data from the BDHS, 2018 and 2022, which was conducted by the National Institute of Population Research and Training (NIPORT), which is a government national research institute researching family planning in Bangladesh and training government officers involved in family planning in Bangladesh, which is situated in Dhaka, Bangladesh. The BDHS is a nationally representative survey that aims to collect demographic and health data and includes a set of indicators of maternal and child health, fertility, family planning, and service utilization that can support the development of related policies. In the data collection process, the person or institution delivering ANC (if any), the number of ANC visits, the date of the first ANC visit, and the components contained in the ANC presented were all recorded. These included blood pressure checks, urine tests for bacteriuria and proteinuria, blood tests for syphilis and anemia, iron supplements, tetanus injections, and antenatal health information. Inquiries regarding health education centered on the spread of knowledge about pregnancy risk indicators, where to address such difficulties, and recommendations to use a qualified birth attendant for delivery. For this analysis, 10,076 (5,012 in 2018 and 5,064 in 2022) women of reproductive age from both the 2018 and 2022 surveys were used to establish the trends in and factors influencing ANC and its quality.

Outcome variable

The primary outcome variables in this study were attendance at four or more ANC visits and attendance at eight or more ANC visits during pregnancy, as recommended by WHO and adopted in recent Bangladesh health guidelines. For each outcome, a binary variable was created: women who attended at least 4 (or 8) ANC visits were coded as "1" (Yes), and those who attended fewer than 4 (or 8) visits were coded as "0" (No). These two binary outcomes were analyzed separately to assess their associated factors.

First, we computed descriptive statistics and 95% confidence intervals. Then, via logistic regression models, we calculated unadjusted odds ratios (ORs) and 95% confidence intervals (CIs) to evaluate the associations between study characteristics and 1) four or more ANC visits, 2) eight or more ANC visits and 3) high-quality ANC.

Study variables

Seven variables were considered for their potential association with attendance at four or more ANC visits and eight or more ANC visits. These were place of birth (urban, rural), women's education (no education, primary, secondary, or higher education), women's work status in the previous 12 months (no, in the previous year, currently working, have a job but have been absent for the last 7 days), the wealth index combined (the DHS wealth quintile is a composite indicator that divides the households into five categories: poorest, poorer, middle, richer and richest; and were derived via principal component analysis on the basis of information from housing characteristics and ownership of household durable goods), religion (Islam, Hinduism, Buddhism, Christianity), unwanted pregnancy when becoming pregnant (then, later, no more), and the husband's education (no education, primary, secondary and higher education).

A variety of factors influence how well prenatal care services are delivered in Bangladesh. In this arrangement, women can receive antenatal care at four locations. They are made up of the public, private, nonprofit, and other sectors. The public or governmental sector included medical college hospitals, district hospitals, upazila hospitals, and community clinics. The private sector included private hospitals, private medical college hospitals, private clinics, and certified doctor chambers. In addition, some nongovernmental organizations (NGOs) provide antenatal care. A doctor, nurse, NGO, family member, and traditional birth attendant were also among the health professionals who offered antenatal care to the women.

Analytical strategy

We initially retained all variables that were significant at $P = 0.25$ in the univariable models. Next, we used a backward elimination approach to remove the model's least essential variable. This step was performed until every variable in the model had a P value of 0.05. We retained the mother's education variable in the final model for the ANC quality model since its significance was somewhat greater than 0.05. We evaluated whether a quadratic component for mothers' ages was still required in both models, and the model with simply a linear term was sufficient in both circumstances. The analyses were carried out via the Statistical Package for Social Sciences (SPSS 26.0).

Results

A total of 10,076 women were included in the analysis, with 5,012 participants from the 2017--2018 period and 5,064 from the 2022 period.

In Table 1, the analysis of 2017–2018 data revealed significant sociodemographic disparities in ANC attendance, with women's education (71% attendance for higher-educated vs 20% for uneducated; aOR=3.37, $p < 0.001$), wealth quintile (richest women having 2.6 times higher odds than poorest), and urban residence (59% vs 42% rural) being the strongest predictors of ≥4 ANC visits. Husband's education (aOR=2.17 for higher-educated) and pregnancy intention (50% attendance for planned pregnancies) were also significant determinants, while religion showed limited association. The same factors influenced ≥8 visits but with more pronounced disparities (22% attendance for higher-educated vs 3% for uneducated), highlighting persistent inequities in maternal healthcare utilization.

In Table 2, the analysis revealed distinct patterns for 4+ versus 8 + visit adherence across sectors. For 4 + visits, private medical colleges showed exceptional performance (aOR=4.45), followed by NGO static clinics (aOR=2.10) and government medical colleges (aOR=2.46), while district hospitals (aOR=1.92) and upazila complexes (aOR=1.36) demonstrated progressively weaker effects. Private clinics (aOR=1.46) and doctor chambers (aOR=1.39) maintained consistent quality advantages. For 8 + visits, these associations attenuated but remained significant, with medical colleges (aOR=2.74) and NGOs (aOR=1.69) retaining leadership. Provider-type analysis showed doctors (4 + aOR=2.21; 8 + aOR=2.22) and nurses (4 + aOR=2.27; 8 + aOR=2.26) delivering equally strong outcomes, while NGO workers showed particularly strong 4 + visit performance (aOR=4.09). Traditional birth attendants had modest 4 + visit effects (aOR=1.66) that became insignificant for 8 + visits, and family-provided care showed no significant benefit at either threshold (4 + aOR=0.95, $p = 0.629$;

Table 1. Characteristics of the study participants who had four or ANC visits, 2017—2018.

4 and more ANC visits					8 and more ANC visits				
**Study Variable**	**Total number of Women**	**Number of women who had 4 or more ANC visits (%)**	**Adjusted OR (95% CI)**	**P value**	**Study Variable**	**Total number of Women**	**Number of women who had 8 or more ANC visits (%)**	**Adjusted OR (95% CI)**	**P value**
**Place of residence**				**<0.001**	**Place of residence**				**<0.001**
Urban	1725	1018 (59.01)	1 (referent category)	1	Urban	1725	297 (17.22)	1 (referent category)	1
Rural	3287	1396(42.47)	.703 (.611 -.808)	<0.001	Rural	3287	287 (8.73)	.615 (.502-.753)	<0.001
**Women's education**				**<0.001**	**Women's education**				**0.001**
No education	312	63 (20.19)	1 (referent category)	1	No education	312	8 (2.56)	1 (referent category)	1
Primary	1392	476 (34.19)	1.833 (1.343 - 2.502)	<0.001	Primary	1392	98 (7.04)	2.595 (1.234- 5.456)	.012
Secondary	2402	1236 (51.45)	2.781 (2.040 –3.793)	<0.001	Secondary	2402	278 (11.57)	3.283 (1.571- 6.861)	.002
Higher	906	639 (71)	3.371 (2.353– 4.807)	<0.001	Higher	906	200 (22.07)	4.177 (1.939- 8.998)	<0.001
**Women's work status in the last 12 months**				**<0.001**	**Women's work status in the last 12 months**				**.334**
No	3022	1475 (48.81)	1 (referent category)	1	No	3022	367 (12.14)	1 (referent category)	1
In the past year	110	51 (46.36)	1.216 (.804- 1.837)	.354	In the past year	110	14 (12.72)	1.228 (.659- 2.288)	.518
Currently working	1865	879 (47.13)	1.360 (1.194- 1.554)	<0.001	Currently working	1865	200 (10.72)	1.188 (.976- 1.445)	.085
Have a job but leave last 7 days	15	9(60)	1.435(.475- 4.336)	.522	Have a job but leave last 7 days	15	3 (20)	1.519 (.393- 5.888)	.345
**Wealth index combined**				**<0.001**	**Wealth index combined**				**.001**
Poorest	1079	333 (3.06)	1 (referent category)	1	Poorest	1079	54 (5)	1 (referent category)	1
Poorer	1017	387 (38.05)	1.245(1.030- 1.505)	.024	Poorer	1017	84 (8.26)	1.546 (1.077- 2.220)	.018
Middle	905	433 (47.58)	1.552 (1.273- 1.832)	<0.001	Middle	905	104 (11.49)	1.790 (1.250- 2.564)	.001
Richer	988	532 (53.85)	1.688 (1.379- 2.087)	<0.001	Richer	988	121 (12.25)	1.544 (1.074- 2.221)	.019
Richest	1023	729 (71.26)	2.606 (2.065- 3.282)	<0.001	Richest	1023	221 (21.60)	2.165 (1.483- 3.160)	<0.001
**Religion**				**.095**	**Religion**				**0.110**
Islam	4589	2179 (47.48)	1 (referent category)	1	Islam	4589	532 (11.53)	1 (referent category)	1
Hinduism	396	218 (55.05)	1.305 (1.043- 1.632)	.020	Hinduism	396	47 (11.87)	.957 (.687- 1.332)	.793
Buddhism	18	11 (61.11)	1.317 (.470- 3.694)	.6	Buddhism	18	1 (5.55)	.382 (.049- 2.999)	.360
Christianity	11	6 (66.66)	1.914 (.445-8.236)	.383	Christianity	11	4 (44.44)	4.986(1.236-20.109)	.024
**Wanted pregnancy when became pregnant**				**<0.001**	**Wanted pregnancy when became pregnant**				**.004**
Then	3954	1985 (50.20)	1 (referent category)	1	Then	3954	498 (12.53)	1 (referent category)	1
Later	651	300 (46.08)	.843 (.705- 1.009)	.062	Later	651	66 (10.14)	.775 (.586- 1.025)	.074
No more	407	129 (31.69)	.614 (.485 -.776)	<0.001	No more	407	20 (4.91)	.494 (.308-.790)	.003
**Husband's education**				**<0.001**	**Husband's education**				**.002**
No education	679	203 (29.89)	1 (referent category)	1	No education	679	39 (5.74)	1 (referent category)	1
Primary	1657	637 (22.15)	1.111 (.906- 1.363)	.310	Primary	1657	118 (7.12)	.946 (.643- 1.389)	.775
Secondary	1635	849 (51.93)	1.407 (1.135- 1.745)	0.002	Secondary	1635	209 (12.78)	1.4 (.953- 2.059)	.087
Higher	962	694 (72.14)	2.169 (1.660- 2.833)	<0.001	Higher	962	212 (22.04)	1.760 (1.146- 2.704)	.010
Don't know	13	5 (38.46)	.907 (.287 – 2.925)	.884	Don't know	13	1 (7.69)	.859 (.107- 6.883)	.885

https://doi.org/10.1371/journal.pone.0337449.t001

Table 2. Factors influencing the quality of ANC services for the 2017--2018 data.

4 and more ANC visits					8 and more ANC visits				
**Study Variable**	**Total number of Women**	**Number of women who had 4 or more ANC visits (%)**	**Adjusted OR (95% CI)**	**P value**	**Study Variable**	**Total number of Women**	**Number of women who had 8 or more ANC visits (%)**	**Adjusted OR (95% CI)**	**P value**
**Place where women receive ANC Govt. sector**					**Place where women receive ANC Govt. sector**				
Medical college hospital	126	84 (66.67)	2.455 (1.667-3.617)	<0.001	Medical college hospital	126	35 (27.78)	2.737 (1.833- 4.087)	<0.001
District hospital	221	132 (59.73)	1.918 (1.439-2.557)	<0.001	District hospital	221	32 (14.48)	1.190 (.808- 1.751)	.378
Upazila health complex	682	342 (50.3)	1.363 (1.139- 1.630)	<0.001	Upazila health complex	682	72 (10.56)	.811 (.624- 1.054)	.117
Community clinic	306	188 (61.44)	1.876 (1.467-2.397)	<0.001	Community clinic	306	40 (13.07)	1.051 (.744- 1.484)	.778
**Private sector**					**Private sector**				
Private hospital	444	240 (54.05)	1.280 (1.044- 1.569)	0.017	Private hospital	444	78 (17.57)	1.883 (1.428- 2.482)	<0.001
Private Medical College	36	29 (80.56)	4.45 (1.939- 10.206)	<0.001	Private medical college	36	7 (19.44)	2.076 (1.230- 4.794)	0.087
Private Clinic	1672	948 (56.69)	1.456 (1.28- 1.657)	<0.001	Private clinic	1672	238 (14.23)	1.493 (1.230- 1.812)	<0.001
Qualified doctor chamber	838	472 (56.32)	1.392 (1.188- 1.632)	<0.001	Qualified doctor chamber	838	123 (14.68)	1.490 (1.185- 1.873)	0.001
**NGO static clinic**	431	295 (68.45)	2.103 (1.701- 2.6)	<0.001	**NGO static clinic**	431	81 (18.79)	1.692 (1.306- 2.192)	<0.001
**Other**	18	11 (61.11)	1.524 (.590- 3.938)	0.385	**Other**	18	0 (0)	.146 (.123- 1.054)	.998
**Health worker who provided ANC**					**Health worker who provided ANC**				
Doctor	1953	1321 (67.64)	2.21 (1.845- 2.655)	<0.001	Doctor	1953	378 (19.35)	2.222 (1.673- 2.952)	<0.001
Nurse	2597	1638 (63.07)	2.266 (1.809- 2.840)	<0.001	Nurse	2597	444 (17.09)	2.259 (1.508- 3.384)	<0.001
NGO	71	46 (64.79)	4.091 (2.483- 6.740)	<0.001	NGO	71	14 (19.72)	4.3 (2.324- 7.958)	<0.001
Relatives	2219	725 (32.67)	.949 (.768- 1.173)	0.629	Relatives	2219	134 (6.04)	1.061 (.719- 1.564)	.767
Traditional birth attendant	536	221 (41.23)	1.658 (1.360- 2.020)	<0.001	Traditional birth attendant	536	43 (8.02)	1.606 (1.113- 2.316)	.011

https://doi.org/10.1371/journal.pone.0337449.t002

8 + aOR=1.06, p = 0.767). These patterns persisted after controlling for socioeconomic factors, suggesting structural advantages of formal healthcare settings are most pronounced for basic (4+) visit adherence.

In Table 3, of the 5,064 women, only 2,065 (41%) attended four or more ANC visits, whereas 403 (8%) of the participants did not attend any ANC visits in 2022. The analysis revealed significant sociodemographic disparities in ANC attendance, with urban women showing higher rates of both 4+ (54.2% vs 34.2% rural; aOR=0.636, p < 0.001) and 8 + visits (7.9% vs 3.6%; aOR=0.838, p = 0.224). Education demonstrated a strong dose-response relationship, where higher-educated women had 2.84 times greater odds of 4 + visits (64.5% vs 20.6% no education; aOR=2.842, p < 0.001) and 2.30 times greater odds of 8 + visits (13.3% vs 1.5%; aOR=2.296, p = 0.140). Wealth disparities were most pronounced, with the richest women having 3.00 times higher odds of 4 + visits (66.9% vs 22.7% poorest; aOR=2.999, p < 0.001) and 5.82 times higher odds of 8 + visits (13.3% vs 1.0%; aOR=5.817, p = 0.001). Husband's education showed similar but attenuated effects (higher education: aOR=2.169 for 4 + , aOR=1.760 for 8 + visits). Notably, while Hindu women had moderately higher 4 + visit rates (aOR=1.377, p = 0.005), religion showed no significant association with 8 + visits, and employment status showed minimal impact after adjustment. These adjusted odds ratios (controlling for all other variables) highlight how economic status and education remain the most powerful predictors of ANC utilization, particularly for intensive (8+) visit schedules, suggesting that financial and educational barriers disproportionately affect comprehensive ANC access.

Table 3. Characteristics of the study participants who had four or more ANC visits, 2022.

4 and more ANC visits					8 and more ANC visits				
**Study Variable**	**Total number of Women**	**Number of women who had 4 or more ANC visits (%)**	**Adjusted OR (95% CI)**	**P value**	**Study Variable**	**Total number of Women**	**Number of women who had 8 or more ANC visits (%)**	**Adjusted OR (95% CI)**	**P value**
**Place of residence**				**<0.001**	**Place of residence**				**<0.001**
Urban	1668	904 (54.19)	1 (referent category)	1	Urban	1668	131 (7.85)	1 (referent category)	1
Rural	3396	1161(34.18)	.636 (.555 -.729)	<0.001	Rural	3396	122 (3.59)	.838 (.631 -.1.114)	0.224
**Women's education**				**<0.001**	**Women's education**				**0.001**
No education	267	55 (20.59)	1 (referent category)	1	No education	267	4 (1.49)	1 (referent category)	1
Primary	1173	316 (26.93)	1.337 (.952 – 1.878)	<0.001	Primary	1173	20 (1.71)	.857 (.282 – 2.606)	.785
Secondary	2666	1076 (40.36)	1.938 (1.388 – 2.705)	<0.001	Secondary	2666	102 (3.83)	1.192 (.409 – 3.476)	.747
Higher	958	618 (64.51)	2.842 (1.958 – 4.127)	<0.001	Higher	958	127 (13.26)	2.296 (.762 – 6.915)	.140
**Women's work status in the last 12 months**				**0.047**	**Women's work status in the last 12 months**				**.608**
No	3769	1563 (41.47)	1 (referent category)	1	No	3769	188 (4.99)	1 (referent category)	1
In the past year	207	76 (31.71)	1.031 (.755 – 1.409)	.847	In the past year	207	8 (3.86)	.990 (.469 – 2.087)	.978
Currently working	1069	414 (38.73)	1.093 (.939 - 1.273)	.251	Currently working	1069	55 (5.14)	1.317 (.946 - 1.832)	.103
Have a job but leave last 7 days	19	12 (63.16)	2.392 (.867 – 6.60)	.092	Have a job but leave last 7 days	19	2 (10.53)	1.944 (.413 – 9.159)	.401
**Wealth index combined**				**<0.001**	**Wealth index combined**				**<.001**
Poorest	1053	239 (22.69)	1 (referent category)	1	Poorest	1053	11 (1.04)	1 (referent category)	1
Poorer	1021	310 (30.36)	1.234(1.007 - 1.512)	.04	Poorer	1021	18 (1.76)	1.364 (.636 – 2.926)	.425
Middle	1008	373 (37.00)	1.420 (1.007 - 1.512)	<0.001	Middle	1008	33 (3.27)	2.109 (1.040 – 4.276)	.038
Richer	997	484 (48.55)	1.969 (1.158- 1.742)	<0.001	Richer	997	60 (6.02)	3.442 (1.740 – 6.811)	.001
Richest	985	659 (66.90)	2.999 (2.380- 3.779)	<0.001	Richest	985	131 (13.29)	5.817 (2.918- 11.596)	.001
**Religion**				**.001**	**Religion**				**0.085**
Islam	4640	1854 (39.96)	1 (referent category)	1	Islam	4640	222 (4.78)	1 (referent category)	1
Hinduism	384	193 (50.26)	1.377 (1.099- 1.726)	.005	Hinduism	384	29 (7.55)	1.375 (.903 – 2.096)	.138
Buddhism	28	12 (42.86)	1.620 (.680- 3.860)	.277	Buddhism	28	2 (7.14)	1.429 (.301 – 6.787)	.653
Christianity	12	6 (50.00)	1.507 (.445 – 5.108)	.510	Christianity	12	0 (0)	0	.999
**Wanted pregnancy when became pregnant**				**0.005**	**Wanted pregnancy when became pregnant**				**.481**
Then	4077	1706 (41.84)	1 (referent category)	1	Then	4077	210 (5.15)	1 (referent category)	1
Later	617	228 (35.95)	.855 (.708- 1.034)	.107	Later	617	29 (4.70)	1.032 (.682- 1.559)	.883
No more	370	131 (35.41)	1.052 (.826 – 1.341)	.679	No more	370	14 (3.78)	1.022 (.551- 1.897)	.944
**Husband's education**				**<0.001**	**Husband's education**				**<0.001**
No education	742	203 (27.36)	1 (referent category)	1	No education	742	10 (1.35)	1 (referent category)	1
Primary	1495	443 (29.63)	.937 (.761 – 1.154)	.540	Primary	1495	31 (2.07)	1.301 (.619 – 2.735)	.488
Secondary	1734	733 (42.27)	1.407 (.924 – 1.409)	.220	Secondary	1734	85 (4.90)	1.4 (.917 – 3.843)	.085
Higher	1037	671 (64.71)	2.169 (1.372 – 2.283)	<0.001	Higher	1037	125 (12.05)	1.760 (1.143- 5.515)	.021
Don't know	9	0 (0)	0	.999	Don't know	9	0 (0)	0	.999

https://doi.org/10.1371/journal.pone.0337449.t003

In Table 4, the quality of ANC received by women is significantly influenced by both the healthcare professional delivering the care and the environment in which it is provided. Women access ANC across four distinct settings: public, private, nonprofit (NGO), and others. Notably, government medical colleges are particularly prominent among public healthcare facilities. In contrast, private hospitals, clinics, and medical colleges play important roles within the private sector. In the government sector, medical college hospitals demonstrated the highest performance for ≥4 visits (53.16% achievement, OR=1.44, p = 0.026), indicating women receiving care there were 44% more likely to meet this threshold compared to other government facilities. However, this advantage did not extend to ≥8 visits (OR=0.99, p = 0.982). District hospitals and Upazila Health Complexes showed no significant associations with ≥4 visits, but the latter exhibited notably reduced odds for ≥8 visits (OR=0.43, p = 0.002), suggesting women at these facilities were 57% less likely to achieve frequent ANC attendance. Community clinics displayed neutral effects for both outcomes. The private sector consistently outperformed government facilities. Private hospitals showed strong positive associations for both ≥4 visits (OR=1.43, p < 0.001)

Table 4. Factors influencing the quality of ANC services for 2022 data.

**4 and more ANC visits**					**8 and more ANC visits**				
**Study Variable**	**Total number of Women**	**Number of women who had 4 or more ANC visits (%)**	**Adjusted OR (95% CI)**	**P value**	**Study Variable**	**Total number of Women**	**Number of women who had 8 or more ANC visits (%)**	**Adjusted OR (95% CI)**	**P value**
**Place where women receive antenatal care Govt. sector**					**Place where women receive antenatal care Govt. sector**				
Medical college hospital	158	84 (53.16)	1.436(1.044 – 1.975)	.026	Medical college hospital	158	9 (5.69)	.992 (.499 – 1.972)	0.982
District hospital	317	131 (41.32)	.883 (.7 – 1.114)	.293	District hospital	317	14 (4.42)	.753 (.433- 1.309)	.314
Upazila health Complex	558	242 (43.36)	.897 (.751 – 1.071)	.228	Upazila health Complex	578	15 (2.59)	.425 (.250 -.722)	.002
Community Clinic	214	105 (49.07)	1.227 (.932 – 1.615)	.145	Community Clinic	214	13 (6.07)	1.105 (.620 - 1.968)	.735
**Private Sector**					**Private Sector**				
Private Hospital	808	388 (48.02)	1.434 (1.211- 1.697)	<0.001	Private Hospital	808	62 (7.67)	2.327 (1.647-3.287)	<0.001
Private Medical College	36	16 (44.44)	1.341 (.691 - 2.601)	0.386	Private Medical College	36	2 (5.56)	1.483 (.349 – 6.296)	0.593
Private Clinic	2053	931 (45.35)	1.296 (1.135- 1.481)	<0.001	Private Clinic	2053	119 (5.79)	1.735 (1.281- 2.351)	<0.001
Qualified Doctor Chamber	510	239 (46.86)	1.347 (1.106- 1.641)	0.003	Qualified Doctor	510	37 (7.25)	2.091(1.406 – 3.109)	0.001
**NGO Static Clinic**	210	121 (57.62)	2.052 (1.539 - 2.735)	<0.001	**NGO Static Clinic**	210	16 (7.62)	2.095 (1.212– 3.621)	0.008
**Other**	30	20 (66.67)	3.014 (1.402 - 6.480)	0.005	**Other**	30	4 (13.33)	3.856 (1.310- 11.35)	.014
**Health worker who provided ANC**					**Health worker who provided ANC**				
Doctor	2823	1479 (52.39)	2.359 (2.009- 2.769)	<0.001	Doctor	2823	214 (7.58)	3.377 (2.178 - 5.238)	<0.001
Nurse	3097	1518 (49.02)	1.433 (1.226- 1.675)	<0.001	Nurse	3097	203 (6.55)	1.126 (.778 – 1.631)	.529
NGO	22	16 (72.73)	8.00 (3.099 – 20.65)	<0.001	NGO	22	1 (4.55)	2.319 (.30 – 17.910)	.420
Relatives	1479	386 (26.09)	.752 (.641-.883)	<0.001	Relatives	1479	28 (1.89)	.634 (.404 -.996)	.048
Traditional birth attendant	40	114 (28.43)	1.251 (.978 – 1.6)	0.075	Traditional birth attendant	401	5 (1.25)	.643 (.251 – 1.647)	.358

https://doi.org/10.1371/journal.pone.0337449.t004

and ≥8 visits (OR=2.33, $p < 0.001$), as did private clinics (OR=1.30 and 1.74 respectively). NGO static clinics demonstrated particularly impressive results for ≥4 visits (OR=2.05, $p < 0.001$), while qualified doctor chambers showed robust effects for ≥8 visits (OR=2.09, $p = 0.001$). The "Other" category, despite small sample sizes, revealed striking ORs of 3.01 ($p = 0.005$) and 3.86 ($p = 0.014$) for ≥4 and ≥8 visits respectively, suggesting exceptional performance in unconventional settings. Provider qualifications significantly influenced ANC attendance. Doctor-led care showed the strongest positive associations, with women 2.36 times more likely to achieve ≥4 visits ($p < 0.001$) and 3.38 times more likely to complete ≥8 visits ($p < 0.001$). Nurses also positively impacted ≥4 visit rates (OR=1.43, $p < 0.001$), though their effect on ≥8 visits was insignificant. NGO health workers demonstrated an extraordinary OR of 8.00 for ≥4 visits ($p < 0.001$), though the wide CI (3.10–20.65) and small sample size warrant cautious interpretation. Conversely, care from relatives was associated with substantially worse outcomes, showing 25% reduced odds for ≥4 visits (OR=0.75, $p < 0.001$) and 37% reduction for ≥8 visits (OR=0.63, $p = 0.048$). Traditional birth attendants showed no significant effects, though their point estimates suggested potentially reduced attendance. The analysis reveals clear disparities in ANC service quality. Private sector facilities consistently outperformed government counterparts, with private hospitals and NGO clinics showing particular strength. Doctor-provided care yielded superior results, while non-professional care (relatives, traditional attendants) correlated with poorer outcomes. Notably, achieving ≥8 visits remained uncommon (≤13.33% across all groups), suggesting systemic barriers to frequent ANC utilization even in optimal settings. These findings underscore the importance of healthcare system factors, particularly facility resources and provider qualifications in shaping ANC service quality and maternal healthcare outcomes.

Table 5 presents data on the quality of ANC received by women during pregnancy in two different time periods: 2017–2018 and 2022. The table compares the percentage of women who had 4 or more visits (≥4 Visits) and 8 or more visits (≥8 Visits) for various study variables. Child's Health Check before discharge declined from 1587 out of 2488 women (63.79%) with ≥4 visits and 430 (17.28%) with ≥8 visits (95% CI: 1.00–1.03) in 2017–2018–1482 out of 2814 (52.67%) and 200 (7.11%) in 2022 (95% CI: 90–95), a drop of 11.12% and 10.17%; Blood Sample taken decreased from 1894 out of 3060 (61.89%) and 494 (16.14%) (95% CI: 65–68) to 1858 out of 3716 (50.00%) and 241 (6.45%) (95% CI: 79–82), a 11.89% reduction; Urine Sample taken fell from 2023 out of 3369 (60.05%) and 534 (15.85%) (95% CI: 72–74) to 1890 out of 3847 (50.86%) and 241 (6.26%) (95% CI: 82–84), a 9.19% decrease; and Mother received Vitamin A saw the sharpest decline, from 1157 out of 2072 (55.84%) and 292 (14.09%) (95% CI: 44–48) to 1262 out of 3086 (40.89%) and 144 (4.67%) (95% CI: 64–68), a 14.95% drop, highlighting a consistent reduction in care frequency potentially linked to access or policy change.

Fig 1 demonstrates a notable reduction in prenatal care (ANC) coverage from 2017–2018 to 2022, with a decreased number of women completing both four or more and eight or more ANC visits in the subsequent survey period.

Table 5. The quality of ANC received during pregnancy in 2017-18 and 2022.

Study variable	2017 -2018				2022			
	Total women	≥4 Visits (%)	≥8 Visits (%)	95% CI	Total women	≥4 Visits (%)	≥8 Visits (%)	95% CI
Iron tablets/syrup taken	3822	2161 (56.54)	533 (13.95)	(.76 -.79)	4013	1851 (46.13)	233 (5.81)	(.78 -.81)
Blood pressure measured	4327	2382 (55.05)	581 (13.43)	(.93 -.95)	4326	2024 (46.79)	248 (5.73)	(.93 -.95)
Blood sample taken	3060	1894 (61.89)	494 (16.14)	(.65 -.68)	3716	1858 (50.00)	241 (6.45)	(.79 -.82)
Urine sample taken	3369	2023 (60.05)	534 (15.85)	(.72 -.74)	3847	1890 (50.86)	241 (6.26)	(.82 -.84)
Mother received vitamin A	2072	1157 (55.84)	292 (14.09)	(.44 -.48)	3086	1262 (40.89)	144 (4.67)	(.64 -.68)
Child's health check before discharge	2488	1587 (63.79)	430 (17.28)	(1.00 – 1.03)	2814	1482 (52.67)	200 (7.11)	(.90 -.95)

https://doi.org/10.1371/journal.pone.0337449.t005

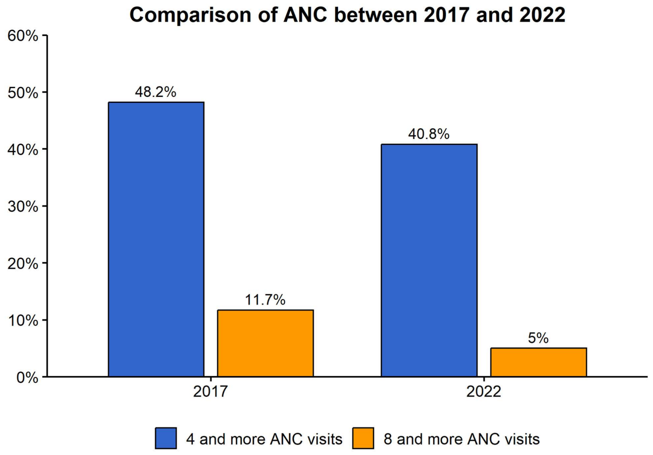

Fig 1. Comparison of ANC between 2017 and 2022.

https://doi.org/10.1371/journal.pone.0337449.g001

Discussion

This study focused on the main features of ANC service delivery and the act of seeking treatment in Bangladesh, in addition to pointing out shortcomings in adhering to ANC subcomponents and striking differences in the ANC landscape of the country. On the basis of the results from the 2017 and 2022 BDHS, only 6.24% of Bangladeshi women who had given birth obtained the required minimum of four ANC visits. Research conducted by Ahinkorah et al. (2022) using data from the 2018 Cameroon Demographic and Health Survey revealed that only 6.3% of women had obtained a minimum of eight prenatal care visits [18]. According to the data obtained from the BDHS (2017--18), 48.2% of women clearly had four or more ANC visits, and from BDHS 2022 data, 41% of women had four or more ANC visits. The report also revealed that 8.14% of women did not receive ANC, whereas 11.7% received eight or more ANC visits. On the other hand, in 2022, 8% of women did not receive ANC, whereas only 5% of women received eight or more visits. Bangladesh requires improvements in the accessibility of appropriate ANC for its female population. This study revealed that certain socioeconomic, demographic, and women's empowerment-related variables had an impact on both the frequency and quality of ANC visits.

Women with more education have a better chance of getting the recommended number of ANC visits and following through with them. Education for women is a key factor in whether or not they use maternal health services, no matter what their socioeconomic status is, how easy it is for them to get healthcare, or what other women have been through [19,20]. In Afghanistan, analogous challenges endure. A recent study revealed that merely 12.7% of Afghan women attended eight or more antenatal care (ANC) visits, with maternal education identified as a significant predictor (AOR = 2.1, 95% CI 1.8–2.5). In Afghanistan, same challenges endure in ensuring adequate usage of prenatal care. A study utilizing data from the Afghanistan Demographic and Health Survey found that a low percentage of women had four or more antenatal care (ANC) sessions. Maternal education emerged as a major predictor of ANC service usage, with women possessing secondary education being more than twice as likely to attend adequate ANC visits compared to those lacking formal education (AOR = 2.43, 95% CI 1.25–4.70) [21]. These results are consistent with our study, which demonstrated a significant correlation between women's education and compliance with WHO-recommended antenatal care visits. Enhancing educational attainment in women has been demonstrated to boost ANC coverage by increasing health awareness and autonomy. In Bangladesh, the completion rate of secondary education for females is quite low, and the adult literacy rate among women is also quite low. This underscores the pressing necessity to prioritize and improve female education in the country [22].

Similar obstacles are present, with the husband's educational attainment being a crucial factor influencing ANC utilization. Women whose husbands completed secondary education were 1.8 times more likely to attend four or more ANC visits and 2.3 times more likely to reach eight visits compared to those whose husbands lacked formal schooling [23]. This pattern aligns with our findings, indicating that the education and involvement of male partners are essential for enhancing maternal healthcare engagement in patriarchal settings.

Women from wealthier families had a greater probability of attending four or more prenatal care appointments, and this correlation also applies to obtaining eight or more ANC visits. Female education and family affordability are the primary factors closely linked to ANC and skilled birth attendance (SBA) in Bangladesh [24]. Female education and family affordability are the strongest predictors of both ANC and SBA in Bangladesh, where four important criteria have been identified: domicile, wealth index, education, and ANC access [25].

Within our study, we pinpointed six noteworthy factors associated with four or more ANC visits, which impact the utilization of antenatal care. These variables included place of residence, wealth index, level of education of the wife and husband, employment status of the woman over the previous year, and intention of the pregnancy. However, in regard to achieving eight or more ANC visits, women's work status within the last 12 months, religion, and pregnancy intentions did not yield significant results. A similar finding was reached in Nepal concerning four or more ANC visits [15].

According to the BNHSM (2017-18), the national strategy guided some principals for maternal care. Service delivery along the continuum of care, strengthening the health system, reducing equalities, community empowerment and engagement, multisector involvement, implementation and impact evaluation are the main strategies used by the Bangladesh government to improve ANC [33]. A developing country such as Bangladesh must focus on promoting awareness of ANC services. There must be at least a 3rd-level maternity hospital (1 per million residents) in every division where complicated pregnancies can be properly treated [34].

Quality

The type of health professional who provides ANC is one of the most important factors influencing the overall quality of ANC that women receive [10]. This study emphasizes the importance of having competent healthcare providers (such as doctors, nurses, or traditional birth attendants) in the process of providing ANC care. Compared with those who were generally less trained (relatives, village health workers, maternal and child health workers, and health assistants/auxiliary health workers), women who attended professional providers had a better chance of obtaining high-quality ANC. This was reflected in the increased percentage of women who received ANC. Women who completed more schooling were more likely to receive high-quality care. According to Ugandan research, women's education is crucial for receiving all of the necessary ANC components [26]. High-quality ANC was more likely to be provided to women from higher-income households. This aligns with research from India and Nigeria, which shows a correlation between better socioeconomic position and a greater likelihood of utilizing all ANC components [27,28]. These relationships may be explained by women with greater socioeconomic status having more affordable health care and access to health information than women with lower socioeconomic status [29]. This study demonstrates how the quality of ANC is impacted by how rural the area where women live is. Compared with women in rural regions, urban women were more likely to obtain high-quality ANC. This could be because access to health care is more challenging in rural locations where there is much less or no transportation infrastructure [30].

Various ANC strategies can be utilized to increase the health of both mothers and infants. Most women in this research had blood pressure measurements, enabling the identification of hypertension, which might indicate the onset of pre-eclampsia [31]. Just over half of the women (67%) had urine tests to detect protein and infection, whereas a slightly larger percentage (61%) had blood samples taken to screen for anemia. These findings are consistent with those of a previous study, which linked poor utilization to health centers' inability to perform such tests [32]. Eighty six percent of women had their blood pressure checked [25]. Iron supplements were used by 74% of the female population. Pregnant women in

impoverished nations who are at greater risk of iron deficiency are advised to take iron supplements [26]. On the other hand, in 2022, the maximum number of mothers with both 4 or more visits and 8 or more visits were concerned with their children. They were concerned with their child's health check before discharge.

This study has several advantages. The survey was a nationwide population-based study conducted with a substantial sample size that accurately represented the whole country. It was carried out meticulously with the assistance of qualified data gatherers and established protocols. The questionnaires were translated and pretested in two regional languages. The poll, however, had several limitations. The information obtained may have been impacted by recall bias due to the self-reported nature of the data and the retrospective design of the study. To mitigate the effects of remembering bias, the study focused on analyzing the most recent pregnancy that occurred during a five-year timeframe prior to the survey. Since the data were obtained from the women themselves, it is uncertain if the healthcare workers executed the procedures optimally.

Quality of ANC comparison (4+ and 8 + visits)

Blood pressure measurement remained the most consistently delivered service (over 80%) during ANC visits, yet the provision of urine and blood tests declined between 2017-18 and 2022 [6,17]. These findings align with Midhet et al. (2025) [19], who reported systemic health service gaps in ANC service delivery in rural Pakistan. Similarly, in India, Nihal and Shekhar (2024) found that underutilization of laboratory tests was largely attributed to supply chain constraints and facility-level inadequacies. [32]

Fifty-five percent of women had their blood pressure checked at 4 + visits, and 13% had their blood pressure checked at 8 + visits; in 2022, blood pressure decreased to 47% and 6%, respectively. [26]. Iron supplements were used by 57% and 14% of the female population at 4+ and 8 + visits, respectively, whereas in 2022, it was used by 46% and 6%, respectively. All expecting women in developing countries who are at high risk of iron insufficiency are encouraged to take iron supplements [25].

Strength and limitation

This study reveals several advantageous components. The survey was a nationwide, population-based study conducted with a substantial sample size that accurately represented the whole country. It was carried out meticulously with the assistance of qualified data gatherers and established protocols. The questionnaires were translated and pretested in two regional languages. The poll, however, had several limitations. The information obtained may have been impacted by recall bias due to the self-reported nature of the data and the retrospective design of the study. To mitigate the impact of remembering bias, we specifically assessed the most recent pregnancy that occurred within a five-year timeframe from the survey. Since the data were supplied by the women, it is uncertain if the healthcare workers executed the procedures optimally. One important limitation of this study is that sampling weights or account for the clustering design inherent in the DHS data. Since the DHS uses a multistage stratified cluster sampling method, failing to incorporate sampling weights and clustering may affect the representativeness of the results and could lead to underestimation of standard errors.

Conclusion

Only 48.2% of Bangladeshi women attended at least four antenatal appointments during their most recent pregnancy, and 11.7% of women attended eight or more antenatal visits in 2017, whereas in 2022, this figure decreased to 41%. However, in 2017 and 2022, only 8% of the women received no ANC during their pregnancy. The socioeconomic parameters associated with having access to high-quality ANC are investigated in this study. These findings support increasing ANC participation among women with lower levels of education and who are socioeconomically disadvantaged to improve results in the short term. However, as part of a plan to promote gender equity and women's empowerment, there must be an emphasis on expanding the education of girls and women in Bangladesh to create long-term advances in women's

access to high-quality ANC. For further improvement, Bangladesh should update the standard to 8 or more ANC visits as a minimum requirement for better maternal health. However, as part of a plan to promote gender equity and women's empowerment, there must be an emphasis on expanding the education of girls and women in Bangladesh to create long-term advances in women's access to high-quality ANC. Mass media campaigns can be broadcast more frequently. This should be carried out in Bangladesh.

Acknowledgments

The authors are grateful to the Demographic and Health Surveys (DHS) Program for providing BDHS data accessibility for conducting the study.

Author contributions

Conceptualization: Sutapa Dey Barna, Md. Akhtarul Islam.

Data curation: Md. Abdul Quayyum, Md. Goffar Hossain.

Formal analysis: Md. Abdul Quayyum.

Investigation: Sutapa Dey Barna, Md. Goffar Hossain, Md. Akhtarul Islam.

Methodology: Sutapa Dey Barna, Md. Abdul Quayyum, Md. Goffar Hossain, Fuad Rahman.

Project administration: Sutapa Dey Barna.

Software: Md. Abdul Quayyum.

Supervision: Sutapa Dey Barna.

Validation: Sutapa Dey Barna, Md. Akhtarul Islam, Fuad Rahman.

Visualization: Sutapa Dey Barna, Md. Abdul Quayyum, Md. Akhtarul Islam.

Writing – original draft: Md. Abdul Quayyum, Md. Goffar Hossain, Fuad Rahman.

Writing – review & editing: Sutapa Dey Barna, Md. Akhtarul Islam.

References

1. Fraser A, Catov JM, Lawlor DA, Rich-Edwards JW. Pregnancy characteristics and women's cardiovascular health. 2018.
2. World Health Organization, UNICEF, United Nations Population Fund, World Bank Group, United Nations Population Division. Trends in maternal mortality 2000 to 2020: Estimates by WHO, UNICEF, UNFPA, World Bank Group and the United Nations Population Division. World Health Organization. 2023. https://www.who.int/publications/i/item/9789240068759
3. GBD 2015 Maternal Mortality Collaborators. Global, regional, and national levels of maternal mortality, 1990-2015: a systematic analysis for the Global Burden of Disease Study 2015. Lancet. 2016;388(10053):1775–812. https://doi.org/10.1016/S0140-6736(16)31470-2 PMID: 27733286
4. Macrotrends. Bangladesh Maternal Mortality Rate 2000-2023. https://www.macrotrends.net/countries/BGD/bangladesh/maternal-mortality-rate. 2023.
5. Odusina EK, Ahinkorah BO, Ameyaw EK, Seidu A-A, Budu E, Zegeye B, et al. Noncompliance with the WHO's Recommended Eight Antenatal Care Visits among Pregnant Women in Sub-Saharan Africa: A Multilevel Analysis. Biomed Res Int. 2021;2021:6696829. https://doi.org/10.1155/2021/6696829 PMID: 34589549
6. Fonzo M, Dalla Zuanna T, Sperotto M, Resti C, Tsegaye A, Azzimonti G, et al. The HIV paradox: perinatal mortality is lower in HIV+ mothers. A case-control study in Ethiopia. European Journal of Public Health. 2020;30(Supplement_5). https://doi.org/10.1093/eurpub/ckaa165.998
7. World Health Organization. WHO antenatal care recommendations for a positive pregnancy experience: maternal and fetal assessment update — imaging ultrasound before 24 weeks of pregnancy. Geneva: World Health Organization. 2022.
8. World Health Organization. WHO recommendations on antenatal care for a positive pregnancy experience. World Health Organization. 2016.
9. World Health Organization. Far more pregnant women getting antenatal care. http://www.who.int/mediacentre/news/releases/2004/pr22/en/. 2004.
10. Abir T, Ogbo FA, Stevens GJ, Page AN, Milton AH, Agho KE. The impact of antenatal care, iron-folic acid supplementation and tetanus toxoid vaccination during pregnancy on child mortality in Bangladesh. PLoS One. 2017;12(11):e0187090. https://doi.org/10.1371/journal.pone.0187090 PMID: 29091923

11. Morgan JL, Nelson DB, Roberts SW, Wells CE, McIntire DD, Cunningham FG. Association of Baseline Proteinuria and Adverse Outcomes in Pregnant Women With Treated Chronic Hypertension. Obstet Gynecol. 2016;128(2):270–6. https://doi.org/10.1097/AOG.0000000000001517 PMID: 27400013
12. Gajate Garrido G. The Impact of Adequate Prenatal Care in a Developing Country: Testing the WHO Recommendations. SSRN Journal. 2011. https://doi.org/10.2139/ssrn.1879464
13. World Health Organization. WHO antenatal care recommendations for a positive pregnancy experience: Maternal and fetal assessment update—imaging ultrasound before 24 weeks of pregnancy. Geneva: World Health Organization. 2022. https://www.ncbi.nlm.nih.gov/books/NBK579604/
14. Rahman SA, Parkhurst JO, Normand C. Maternal Health Review Bangladesh. Health Systems Development Programme, Policy Research Unit (PRU), Ministry of Health and Family Welfare, Government of the People's Republic of Bangladesh. 2003. https://assets.publishing.service.gov.uk/media/57a08cf4ed915d622c0016a7/02-03_bangladesh.pdf
15. Joshi C, Torvaldsen S, Hodgson R, Hayen A. Factors associated with the use and quality of antenatal care in Nepal: a population-based study using the demographic and health survey data. BMC Pregnancy Childbirth. 2014;14:94. https://doi.org/10.1186/1471-2393-14-94 PMID: 24589139
16. Pattinson RC. Are deaths due to prematurity avoidable in developing countries?. Trop Doct. 2004;34(1):7–10. https://doi.org/10.1177/004947550403400104 PMID: 14959961
17. Tuladhar H, Dhakal N. Impact of Antenatal Care on Maternal and Perinatal utcome: A Study at Nepal Medical College Teaching Hospital. Nepal j obstet gynaecol. 2012;6(2):37–43. https://doi.org/10.3126/njog.v6i2.6755
18. Ahinkorah BO, Seidu A-A, Budu E, Mohammed A, Adu C, Agbaglo E, et al. Factors associated with the number and timing of antenatal care visits among married women in Cameroon: evidence from the 2018 Cameroon Demographic and Health Survey. J Biosoc Sci. 2022;54(2):322–32. https://doi.org/10.1017/S0021932021000079 PMID: 33632369
19. Chakraborty N, Islam MA, Chowdhury RI, Bari W, Akhter HH. Determinants of the use of maternal health services in rural Bangladesh. Health Promot Int. 2003;18(4):327–37. https://doi.org/10.1093/heapro/dag414 PMID: 14695364
20. Lincove JA. Growth, Girls' Education, and Female Labor: A Longitudinal Analysis. JDA. 2008;41(2):45–68. https://doi.org/10.1353/jda.2008.0023
21. Yeo S, Bell M, Kim YR, Alaofè H. Afghan women's empowerment and antenatal care utilization: a population-based cross-sectional study. BMC Pregnancy Childbirth. 2022;22(1):970. https://doi.org/10.1186/s12884-022-05328-0 PMID: 36575408
22. Mahmud S, Amin S. Girls' Schooling and Marriage in Rural Bangladesh. Research in the Sociology of Education. Emerald (MCB UP):71–99. https://doi.org/10.1016/s1479-3539(06)15004-1
23. Simkhada B, Teijlingen ER van, Porter M, Simkhada P. Factors affecting the utilization of antenatal care in developing countries: systematic review of the literature. J Adv Nurs. 2008;61(3):244–60. https://doi.org/10.1111/j.1365-2648.2007.04532.x PMID: 18197860
24. Bhowmik J, Biswas RK, Woldegiorgis M. Antenatal care and skilled birth attendance in Bangladesh are influenced by female education and family affordability: BDHS 2014. Public Health. 2019;170:113–21. https://doi.org/10.1016/j.puhe.2019.02.027 PMID: 30991173
25. World Health Organization. Antenatal care in developing countries: Promises, achievements and missed opportunities. World Health Organization. 2004.
26. Edward B. Factors influencing the utilisation of antenatal care content in Uganda. Australas Med J. 2011;4(9):516–26. https://doi.org/10.4066/AMJ.2011.849 PMID: 23393544
27. Pallikadavath S, Foss M, Stones RW. Antenatal care: provision and inequality in rural north India. Soc Sci Med. 2004;59(6):1147–58. https://doi.org/10.1016/j.socscimed.2003.11.045 PMID: 15210087
28. Babalola S, Fatusi A. Determinants of use of maternal health services in Nigeria--looking beyond individual and household factors. BMC Pregnancy Childbirth. 2009;9:43. https://doi.org/10.1186/1471-2393-9-43 PMID: 19754941
29. Neupane S, Doku DT. Determinants of time of start of prenatal care and number of prenatal care visits during pregnancy among Nepalese women. J Community Health. 2012;37(4):865–73. https://doi.org/10.1007/s10900-011-9521-0 PMID: 22134620
30. V Teijlingen E, Simkhada P, Stephens J, Simkhada B, Rogers S, Sharma S. Making the best use of all resources: developing a health promotion intervention in rural Nepal. Health Renaissance. 2012;10(3):229–35. https://doi.org/10.3126/hren.v10i3.7141
31. World Health Organization. WHO antenatal care randomized trial: Manual for the implementation of the new model. WHO program to map best reproductive health practices. 2002.
32. Osungbade K, Oginni S, Olumide A. Content of antenatal care services in secondary health care facilities in Nigeria: implication for quality of maternal health care. Int J Qual Health Care. 2008;20(5):346–51. https://doi.org/10.1093/intqhc/mzn026 PMID: 18621778
33. Government of the People's Republic of Bangladesh Ministry of Health and Family Welfare. Bangladesh National Strategy for Maternal Health 2019-2030.
34. Directorate General of Health Services. Bangladesh national strategy for maternal health 2019-2030. Ministry of Health and Family Welfare, Government of the People's Republic of Bangladesh. 2019. https://dghs.portal.gov.bd/sites/default/files/files/dghs.portal.gov.bd/page/b854defc_b4ca_4a77_b6de_1f8cfcc281c2/2025-04-10-10-53-bad53858a0ac0faccce06c86d7a65f77.pdf
